# Artificial Intelligence (AI) in Detection of Abiotic Stress in Plants: A Review

**DOI:** 10.3390/s26041122

**Published:** 2026-02-09

**Authors:** Anushree Matabber, Lionel Lami-Ndame Rhuhanga, Shinsuke Agehara, Maryam Mozafarian

**Affiliations:** 1 Department of Vegetable and Mushroom Growing, Hungarian University of Agriculture and Life Sciences, 1118 Budapest, Hungaryrhuhanga.lami.ndame@stud.uni-mate.hu (L.L.-N.R.); 2 Faculty of Agriculture and Environmental Sciences, Université Catholique de Bukavu, Bukavu B.P. 285, Democratic Republic of the Congo; 3 Gulf Coast Research and Education Center, Institute of Food and Agricultural Sciences, University of Florida, 14625 CR 672, Wimauma, FL 33598, USA; sagehara@ufl.edu

**Keywords:** artificial intelligence, abiotic stress, precision agriculture, machine learning, deep learning, imaging techniques

## Abstract

Global agriculture is facing significant threat from climate-driven abiotic stress, which endangers global food security by impacting crop performance and adaptation. However, traditional abiotic stress detection methods are often labor-intensive and lack precision and scalability. Efficient and reliable solutions are needed to meet rising global food demand. Recent advances in artificial intelligence (AI) offer highly accurate, non-invasive, and sustainable approaches for abiotic stress detection. This paper reviews the impact of AI, and specifically Machine and Deep Learning algorithms, coupled with synergistic technologies and diverse datasets (imaging techniques and Internet of Things (IoT) infrastructures), to identify unique signatures of abiotic stress, and assess its impact on growth and physiological performance. It contrasts with other reviews that address individual technologies and algorithms, while presenting abiotic stress detection as a secondary objective. We examined peer-reviewed journal articles on the use of AI in detecting abiotic stress. The reviewed literature was chosen based on the stress category, sensing mode, and AI technologies employed. A comparative analysis was performed to explore potential advancements of AI-based abiotic stress detection methods over traditional approaches and also challenges lied to the adoption of AI in agriculture for abiotic stress detection.

## 1. Introduction

Current global trends highlight the exponential growth of the world’s population, which today stands at around 8 billion [1]. This situation is increasingly alarming as it places tremendous pressure on global food supply that relies heavily on plant-based food [1]. However, food production is constantly affected by environmental changes, which constitute the main barrier to supplying sufficient, nutritious, and safe food to feed the world population [2,3]. With the population projected to surpass 10 billion by 2050, food availability will become even more critical, while agricultural productivity may decline due to the cumulative impacts of climate change [4].

Climate change manifests through multiple environmental factors, including heat, drought, soil salinity, nutrient deficiency, heavy metal contamination, flooding, and cold [5,6], collectively threatening global agriculture [7]. Altered precipitation patterns often result in more intense rain events with longer dry periods, reducing water availability for agriculture, while also causing soil erosion and nutrient leaching, which hinder crop growth and yield. Crop production in areas affected by poor precipitation in Africa and South Asia is expected to decline by approximately 25% by 2080 [8]. Prolonged drought seasons intensify heat stress and water scarcity, leading to poor crop performance in terms of both yield and quality, and in severe cases, crop mortality [9]. Drought stress decreases the average crop yields by approximately 50% [8]. Rising temperatures further create favorable conditions for pests, enhancing their survival and reproduction rates [9]. Additionally, climate change drives extreme weather events that devastate crops, damage habitats, and undermine safe farming environments. Floods, for example, destroy crops, erode soil, and disrupt ecosystems, rendering farmland unsuitable for cultivation. Other climate change impacts include increased soil salinity from rising sea levels, degradation of soil health and microbial balance, and biodiversity loss [10]. These consequences are particularly severe in vulnerable regions such as Sub-Saharan Africa, Southeast Asia, and island nations in the Pacific Ocean [11]. For example, statistical data on wheat and maize production in East Africa is expected to decline by 15% and 40%, respectively, due to the combined effects of abiotic stress factors stemming from climate change. Globally, the impacts of abiotic stress factors due to climate change are expected to hit USD 23 billion annually by 2050 [8].

Early detection of abiotic stress is crucial for designing effective interventions to maintain crop health, yield, and quality [12]. Traditionally, abiotic stress detection relied on manual methods like field scouting, visual diagnostics, and laboratory testing. These methods involve visually assessing plant health, stature, and production, as well as destructive sampling to evaluate shoot-to-root ratios, fruit or grain weights, and biomass, which are labor-intensive, destructive, and time-consuming, especially over large areas [13,14].

Recent technological advancements have led to the development of sensor-based approaches that leverage artificial intelligence (AI) for non-destructive, early stress detection with improved accuracy, efficiency, and scalability [14,15]. In particular, the integration of machine learning (ML) and deep learning (DL) with the Internet of Things (IoT) has enabled faster, more precise, and non-invasive stress diagnosis using remote sensing (RS) tools, such as RGB cameras, infrared sensors, fluorescence imaging, thermal cameras, multispectral and hyperspectral imaging tools [16]. It also allows more efficient abiotic stress detection by capturing subtle phenotypic changes during early stress development [17]. Among these tools, RGB imaging, which utilizes red (R), green (G), and blue (B) spectral bands, has been widely studied and used for stress detection in plants [18,19,20]. In addition, spectral (multispectral and hyperspectral), thermal, and fluorescence imaging technologies have shown great potential in improving stress detection precision. For example, thermal imaging detects infrared radiation emitted from plant surfaces to visualize temperature variations in plants, providing insights into their physiological stress status [21,22]. Fluorescence imaging measures chlorophyll *a* fluorescence emitted by photosynthesis II in chloroplasts, enabling sensitive stress detection based on the efficiency and functionality of photosynthesis [17,23]. Multispectral and hyperspectral spectral imaging measure light reflectance from plat surfaces across a broad range of wavelengths, including visible, near-infrared, and infrared regions, to detect biochemical and physiological changes associated with abiotic stresses, such as drought, heat, and nutrient deficiency [21].

By analyzing data collected from these sensors, ML and DL algorithms detect patterns and develop accurate predictions for plant stress detection. ML techniques, such as Support Vector Machines (SVM) and Artificial Neural Networks (ANN), analyze plant image data to learn from experience and accurately identify stress-related symptoms in real time. By contrast, DL, a subfield of ML that employs Deep Neural Networks (DNNs), is particularly effective for processing complex, high-dimensional datasets and has become a preferred approach for stress identification [18].

This review synthesizes current advances in AI-based detection of plant abiotic stress, highlighting key sensing technologies, ML and DL algorithms, and their integration across spatial and temporal scales. We raise the role and applications of AI in agriculture by highlighting the most relevant ones. We compare traditional and AI-based approaches, summarize state-of-the-art tools for specific stress types, and discuss challenges and future directions for scalable field applications.

## 2. Roles of Artificial Intelligence in Agriculture

Technology is rapidly revolutionizing diverse sectors, from healthcare and urbanization to education and tourism, and agriculture is no exception. As a vital sector for sustaining life, agriculture is undergoing a profound technological revolution. According to the World Economic Forum [24], agriculture contributes nearly 25% of the global greenhouse gas emissions. Meanwhile, the global population is expected to increase by approximately 18.3%, from 8.2 billion to 9.7 billion, by 2050. Consequently, global food demand will increase sharply, with crop-based food consumption expected to be substantially greater than the population by 2050 [25].

While agriculture is often cited as a major contributor to climate change, it is also among the most affected sectors, facing challenges such as water scarcity, changing climate patterns, and diminishing availability of arable land. There is an urgent need to find sustainable ways of increasing agricultural productivity while minimizing greenhouse gas emissions. AI is increasingly recognized as a key tool for promoting sustainable agricultural practices. The World Economic Forum [24] recently projected the prevalence of AI in agriculture to increase from USD 1.7 billion in 2023 to USD 4.7 billion in 2028. AI in agriculture opens doors to innovations that optimize the use of resources, minimize waste, improve productivity, and promote sustainability [26,27]. AI is at the core of smart agriculture, providing the analytical and decision-making capability that transforms data into actionable insights.

**Figure 1:**
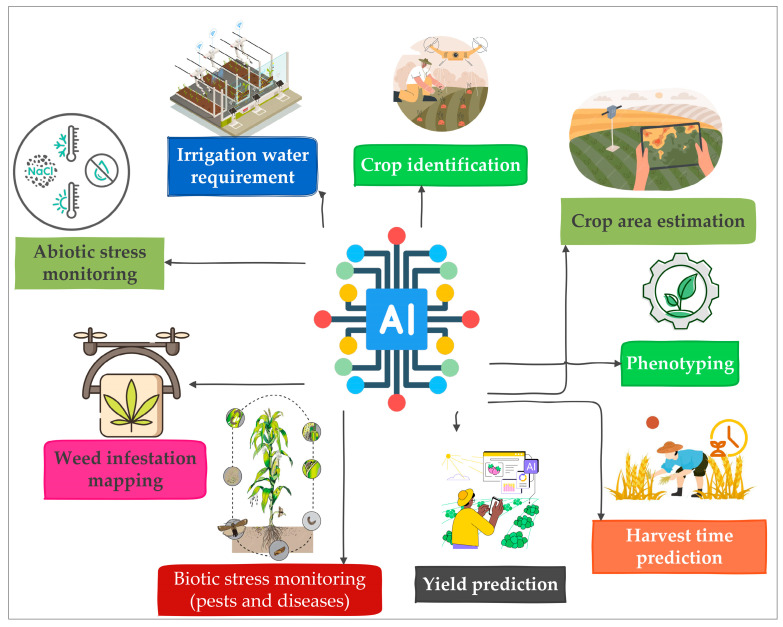
Application of artificial intelligence in agriculture.

Agriculture ranks among the world’s top three labor-intensive sectors due to routine practices that rely heavily on manual labor across farm management, post-harvest handling, marketing, and logistics. The growing demand requires a major leap in production, which calls for extra labor. AI and complementary advanced technologies, such as IoT, ML, and DL, are revolutionizing agriculture through automation, enhancing production efficiency and minimizing labor demands. Smart farm machinery designed for irrigation, harvesting, and other farm operations has become more efficient, less time-consuming, and less labor-dependent [28]. The applications of AI in agriculture are extensive, as illustrated in Figure 1.

AI technologies are advancing modern agriculture by improving soil health management, seed quality assurance, weather prediction, diseases, pest and weed control. During the pre-production phase, AI enhances soil assessment by analyzing properties such as moisture, nutrient availability, and other chemical and physical properties using technologies like ML and RS [26]. These technologies provide accurate, real-time data to guide irrigation scheduling, fertilizer management, and soil conservation practices [29,30].

In seed quality assurance, AI replaces traditional manual inspections with automated data-driven analyses. ML and predictive modeling evaluate seed traits, simulate environmental conditions, and identify varieties best suited to specific climates [31,32]. This promotes efficiency, accuracy, and sustainability by enabling farmers to access high-quality, resilient seeds while optimizing planting schedules and resource use [31]. AI also revolutionizes weather prediction by analyzing climate data from satellites and sensors to forecast rainfall, temperature, and extreme events with greater accuracy than traditional observation methods [33,34]. Reliable forecasts help farmers plan production schedules and reduce risks associated with climate variability. In managing biotic stress, AI-driven systems detect and classify pests and diseases using image recognition and environmental data [35,36]. These technologies provide early warnings and suggest targeted control measures, minimizing pesticide use and promoting sustainable pest management [28,37]. Similarly, AI-powered DL models distinguish weeds from crops, allowing drones and automated systems to apply precise, minimal interventions [38,39,40]. This reduces herbicide pollution, enhances soil and environmental health, and improves overall crop productivity. AI has critically elevated the burden of disease infection in plants through accurate and rapid automated disease detection. AI-based abiotic stress detection deploys models that can detect elements which are regularly difficult to analyze, such as leaf development, leaf pigmentation, pod structure, and growth rate [29]. Early crop health data and disease location enhance the development of comprehensive disease control plans which enhance crop yield and quality [30]. A study conducted by Trivedi et al. [31] demonstrated that Google Colab, a convolutional neural network, showed 98.49% effectiveness in detection of tomato leaf disease through evaluation of over 300 images of tomato leaves. Furthermore, advanced studies have tried to distinguish symptoms caused by abiotic and biotic stress by identifying unique spectral fingerprints using hyperspectral imaging and AI models. For example, a case study conducted by Navarro et al. [32] compared the effects of fungal diseases such as *Fusarium* and *Rhizoctonia*, against abiotic factors like salinity and drought by analyzing unique light reflectance patterns of leaves of Wild rocket (*Diplotaxis tenuifolia*) which helped to identify specific spectral signatures that distinguish different types of damage on the plant. To classify these stresses, a trained artificial neural network combined with hyperspectral imaging were combined and performed high classification accuracy and distinguished both stress types.

Overall, the integration of AI in agriculture enhances precision, sustainability, and resilience by enabling data-driven decision-making across all stages of production.

## 3. Comparison of Traditional and AI-Based Methods for Plant Abiotic Stress Detection

Traditional methods for detecting plant abiotic stress rely primarily on field scouting, visual assessments, and routine nutrient monitoring practices. Field scouting remains the most common approach used by growers and crop advisors because it offers simplicity and real-time diagnosis. However, visual assessments are inherently subjective, highly dependent on scouts’ experience, and typically detect stress only after symptoms become apparent. In addition to visual scouting, plant tissue nutrient analysis is widely used to monitor plant nutritional status and diagnose nutrient-related stress. This method involves collecting multiple leaves or petioles from representative plants, often forming a composite sample from a defined management unit such as 8 to 20 hectares, and submitting the samples to commercial or university analytical laboratories for macro- and micro-nutrient quantification. Similarly, soil sampling is routinely conducted prior to field preparation to assess baseline soil nutrient levels, pH, salinity, and other chemical properties necessary for determining appropriate fertilization rates. While these laboratory-based diagnostic tools provide high accuracy and well-established interpretive guidelines, they can be labor-intensive, require careful sampling protocols, and incur analytical costs that limit sampling frequency during the season. Some growers also rely on several physiological tools to complement visual and laboratory-based assessments. SPAD meters can be used to measure relative chlorophyll content as an indicator of nitrogen status or overall leaf health, offering rapid, non-destructive, and objective assessments. Other handheld devices may also be used. For example, infrared thermometers allow quick spot measurements of canopy temperature to detect heat or water stress, while simple soil moisture meters or tensiometers help identify water stress. These tools provide immediate, quantitative measurements but can be limited by their point-based nature, susceptibility to environmental variability, and the need for consistent measurement protocols to ensure reliable interpretation across fields and seasons.

AI-based methods for detecting abiotic stress differ fundamentally from these traditional approaches by leveraging RS technologies and computational models to continuously monitor plant or environmental conditions and identify subtle stress signatures before visible symptoms occur. These systems commonly use imaging platforms, such as multispectral, hyperspectral, thermal, fluorescence, or high-resolution RGB sensors, mounted on Unmanned Aerial Vehicles (UAVs), ground-based vehicles, or stationary proximal units. The general procedure involves collecting spatially and temporally rich datasets, preprocessing images to correct for illumination or sensor noise, extracting stress-related features such as vegetation indices or canopy temperature, and training ML or DL models to classify or predict stress conditions. These AI-driven systems offer significant advantages, including non-destructive and high-throughput monitoring, early detection of pre-symptomatic stress, and the ability to generate spatially explicit stress maps across entire fields or production regions. However, AI-based approaches also present challenges. They require large, high-quality labeled datasets for model training, consistent sensor calibration across seasons, and robust computational workflows to ensure reliable performance. Model transferability can be limited when environmental, varietal, or management conditions differ from those used during training. Additionally, the acquisition and maintenance of advanced sensors, data infrastructure, and analytical expertise may increase operational costs and create barriers to adoption for resource-limited growers. Despite these constraints, AI-based approaches provide powerful decision-support tools with the potential to transform stress monitoring and enable precision crop management when effectively integrated into commercial production systems.

AI is more effective and accurate in the detection of plant stress as compared to traditional methods. Traditional methods of stress detection in plants include physical observation of plant morphology, use of hand-held devices such as mobile phones and porometers, and laboratory analysis. While these methods have been convenient in the recent past, they pose several disadvantages. Traditional methods are time-consuming; hence, stress detection may be delayed, eventually delaying the implementation of necessary interventions. Physical observation of plant features is limited to elements observable using the human eye in visible light, hence hindering early detection of stress in plants. These methods are destructive and require extensive labor. The intensity of labor increases with an increase in the size of land and intensity of work, thereby increasing the costs of production. These disadvantages make it difficult to scale the application of traditional methods in larger tracts of land. There is a need for scalable and cost-effective solutions that enhance accuracy without compromising optimal crop production [33].

AI techniques provide more accurate and effective solutions. ML and DL models confer high accuracy in systems by using data-driven decisions. The data collected is accurate and detailed [34]. For example, imaging sensors such as spectral and fluorescence using specific wavelengths to study leaf structure and evaluate physical and biochemical properties relevant to the abiotic stress factors under study. These systems align observations of changes in physical appearances with biochemical changes to confirm vulnerability and plant response to various abiotic factors [35]. This evaluation enhances the accuracy of results, hence increasing precision in decision-making. The accuracy of AI tools is not static. It is progressively getting better due to the increased availability of high-quality data on environmental changes and advancements in phenotyping plants and their response to different abiotic factors. As such, AI provides highly scalable solutions that can be applied on a broad scale in agriculture, ranging from localized applications using UAVs to global applications using satellite imagery [18].

Table 1 summarizes the key comparison features of traditional methods versus AI-based methods in abiotic stress detection by emphasizing the key features in detection timing, precision and accuracy, scalability, labor efficiency and disadvantages of both methods.

## 4. AI Basics and How They Work: Machine Learning and Deep Learning

### 4.1. Relation Between Artificial Intelligence Machine Learning and Deep Learning

AI is a field of computer science that uses data-driven algorithms, such as ML and DL, to enable computers to learn from data and perform tasks that traditionally require human intelligence. Its main goal is to learn, reason, and perceive similarly to human beings to enhance automation. ML is a subset of AI that learns from exposure to mimic human intelligence and behavior to perform tasks independently, depending on the data provided. DL is a subset of ML that enhances learning from data using neural networks [36]. These technologies use data to provide solutions to current problems and predict future outcomes. It also uses data to understand how systems work and automate processes using algorithms to minimize labor requirements without compromising on efficiency [37].

ML algorithms are developed through supervised, unsupervised, and reinforcement learning. Supervised learning occurs when systems are exposed to labeled data, while unsupervised learning occurs when systems are exposed to unlabeled data but can track patterns and develop insights [38].

In the supervised learning two fundamental tasks can stand out in particular: classification and regression tasks [39]. The classification aims to predict discrete values by using different algorithms such as Logistic Regression, Decision Trees, Support Vector Machines (SVM), k-Nearest Neighbors (k-NN), Naive Bayes, Random Forest (RF) [14]. The regression tasks deal with predicting continuous numerical values [14] and utilize different algorithms such as Regression, SVM Regression and Neural Network Regression [39].

The unsupervised learning is categorized into three algorithms clustering, association and dimension reduction. The most common algorithms in the category are K-means used in clustering and Principal Component Analysis (PCA) used in dimension reduction [40].

Reinforcement learning occurs when machines can leverage on data available to make decisions relevant to the subject and context [14,38]. They utilize diverse algorithms such as Q-Learning, Deep Q-Networks (DQN), Policy Gradient Methods, Temporal Difference (TD), Monte Carlo, etc. [14].

**Figure 2:**
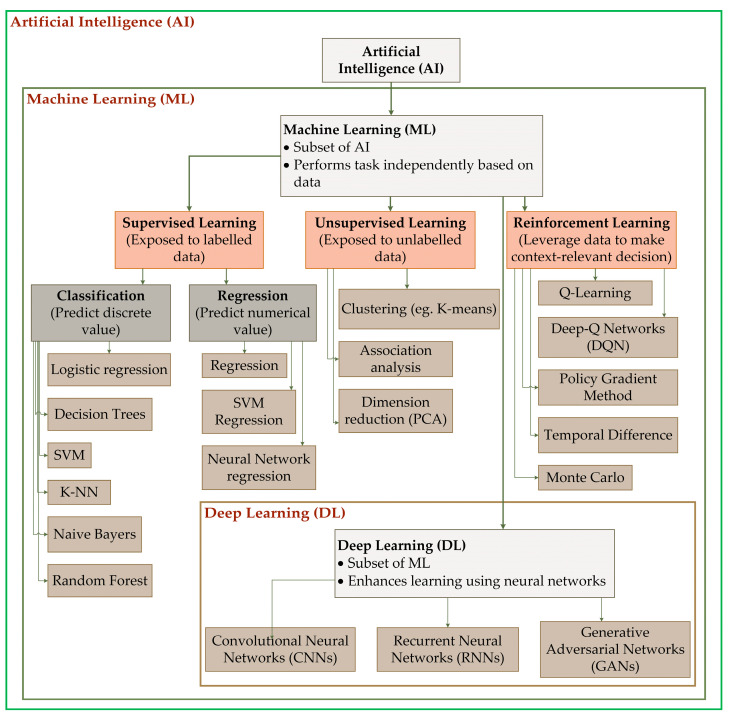
Relation between artificial intelligence, machine learning, and deep learning.

DL, a subcategory of ML, has shown advanced and powerful data treatment application in agriculture due to its complex neural networks which allow the algorithms to treat complex problems. The DL architecture comprise three main models which have been widely used in agriculture Convolutional Neural Networks (CNNs) [20,41], Recurrent Neural Networks (RNNs), and Generative Adversarial Networks (GANs) [14]. This explains how machines can follow instructions, act predictably, or make decisions during automation using AI. Figure 2 shows the relation between AI, ML and DL.

### 4.2. Data Collection, Preprocessing and Model Training

#### 4.2.1. Data Collection

Data for AI-based abiotic stress detection are typically acquired across a different range of spatial scales spanning from individual leaf measurements to general observations. They are generally categorized based on the platforms used for data collection. At proximal and leaf scale, data are captured at a very focalized level using onsite devices such as digital camera, smartphones, handheld spectrometers and raspberry pi-powered sensors for identifying subtle biochemical and structural changes in leaves [18,42,43]. At a sub-field scale, data are acquired using high-resolution UAVs for field scale stress mapping [43]. And finally, at a macro-scale level by using satellites for regional monitoring [18,43]. These platforms rely on the use of imaging sensors like RGB for color and texture analysis, multispectral and hyperspectral cameras for detecting subtle biochemical and physiological changes in the chlorophyll and water content across narrow wavelength bands, thermal infrared sensors for measuring stress-induced leaf temperature and fluorescence imaging to monitor real-time photosynthetic efficiency [18,44]. Structural data are further augmented by LiDAR systems that utilize laser pulses to build 3D point clouds of the canopy architecture and leaf area index [18]. It is important to capture high quality images of plants based on appropriate imaging equipment for a successful abiotic stress detection based on computer vision. Different conditions and adjustments of image might be respected and typically linked to conditions like exposure time, aperture, focal length, resolution and image background [42]. The biological data acquired via image techniques is contextually integrated with environmental variables acquired through IoT-enabled sensors, which constantly record atmospheric parameters such as temperature, relative humidity, alongside with soil parameters such as volumetric water content and nutrient levels [45].

#### 4.2.2. Preprocessing Steps

Once data is collected, it is processed to meet the machine’s specifications for easy model training [13]. However, a universal process of data preprocessing may be difficult to realize due to variation in format types of imaging sensors utilized for data collection [13]. The initial phase of the preprocessing step typically involves radiometric calibration and atmospheric correction to transform digital numbers into surface reflectance, alongside geometric correction and ortho-rectification to account for topographic influences and Earth curvature in case of optical satellite images [43]. For the success of this workflow, it is important to implement sensor-specific corrections, which involve a range of essential adjustments such as modifying emissivity values and compensating for ambient influences on thermal data, rectifying the spectral smile effect observed in hyperspectral cubes, and performing dark and white reference points for effective spectral normalization [44]. To ensure that models are compatible and that computations are performed efficiently, the datasets are subjected to a process of image resizing and standardization through nearest neighbor interpolation or resampling, often enhanced by targeted cropping or image segmentation to isolate plant architecture from background noise such as soil and shadows [42]. Noise reduction is another important step which might follow. The noise reduction step uses Median or Gaussian filter and Savitzky–Golay smoothing, and simultaneously, color space conversion which converts RGB images to other representations such as L*a*b color space (where L is the lightness number, a is the range between green and red, and b is the range between blue and yellow), or HSI (Hue, Saturation and Intensity) which aim to enhance the intensity and detect subtle physiological signatures [44]. Data normalization and standardization, such as Min-Max scaling or Z-score normalization, are then applied to facilitate faster gradient descent convergence and reduce computing costs [42]. To manage high-dimensional data, particularly from hyperspectral sensors, Principal Component Analysis (PCA) is frequently used for dimensionality reduction while minimizing information loss [44]. Finally, the process includes feature extraction to convert raw signals into descriptive representations such as Vegetation Indices (VIs) in order to mitigate data scarcity and class imbalances [44].

#### 4.2.3. Model Training

Model training is a confirmatory stage in AI abiotic stress detection and diagnosis. It involves ensuring model effectiveness in AI detection. Training merges data-driven inputs with real-world performance to project the compatibility between the model and varying environments. Training also enhances model ability to identify, categorize, and evaluate the intensity of the stress. This is achieved by randomly using different datasets to train and test model performance. It is also evaluated by assessing model performance in different environments [46]. Real-word tests ensure reinforcement of multitask learning by enhancing reception of varying plant signals that denote different stress factors. Multitask learning ensures early stress detection, diagnosis, and decision-making, hence improving crop production and eventual food safety and security.

The evaluation of AI models involves a multi-dimensional process that integrates statistical performance metrics. For stress classification tasks, models are assessed using overall accuracy and the Kappa coefficient, and reinforced by class-sensitive metrics like Precision, Recall, F1-score, and balanced accuracy to account for inherent class imbalances where healthy samples significantly outnumber stressed ones [43,47].

## 5. Available Tools in AI for Abiotic Stress Detection in Plants

### 5.1. Internet of Thing Technologies

Various AI tools are used in detecting abiotic stress in plants, including IoT sensors, UAVs, RS, and predictive analytics. The IoT technology is used in abiotic stress detection in plants for data collection and automation. IoT sensors integrated in different systems monitor designated variables in real time, enhancing data collection and transmission (Table 2). This technology collects accurate data on crop demands and soil properties, which are used in developing data-driven precision farming strategies and automating critical processes to minimize labor demand and time wastage [48]. IoT is applicable in abiotic stress detection through real-time monitoring of abiotic factors such as temperature, humidity, and soil moisture content. IoT may also be applied to monitor the emission of greenhouse gases to mitigate the exacerbation of the climate change crisis [49]. In farming, IoT sensors are mounted on machinery such as tractors, where they collect real-time data and enhance automation. For example, tractors with global positioning system sensors can plant seeds and apply fertilizer autonomously. IoT mounted on UAVs captures real-time aerial images from which information is extracted by DL models to assess crop health. IoT holds massive potential to steer climate-smart agriculture through sustainable practices such as precision farming [50].

### 5.2. Remote Sensing-Based Technologies

RS platforms present another critical tool in abiotic stress detection in plants. There are different types of RS technologies which include proximal ground-based data for detailed physiological monitoring, UAVs/Drones for field scale assessment, and satellites-based RS for regional surveillance and non-invasive detection of abiotic stress such as water, drought, salinity, heat, and nutrient deficiency stress [72,76,77,78], by using technologies like RGB, multispectral, hyperspectral and thermal infrared sensing and fluorescence sensing [72]. Spectral data is converted into vegetation indices (VIs) that quantify plant properties and stress level. The most common VIs used in stress detection include NDVI (Normalized Difference Vegetation Index) which detects drought/nutrient stress, NDWI (Normalized Difference Water Index) which detects drought stress, EVI (Enhanced Vegetation Index) for general stress, PRI (Photochemical Reflectance Index) for heat/light/nutrient stress, LST (Land Surface Temperature) for heat/drought stress, CWSI (Crop Water Stress Index), and NDDI (Normalized Difference Drought Index) in drought stress detection [76].

The proximal ground-based RS offers a powerful approach in plant stress detection and constitutes a high-resolution sensing tool which can be placed at various depths from the plant target by utilizing both portable and fixed spectrometers to capture spectra at leaf and canopy scales [75]. For collecting data, different sensor types are used, including RGB imaging, hyperspectral imaging, multispectral imaging, thermal infrared radiometry, sun-induced chlorophyll fluorescence, and LiDAR [72].

UAVs-based RS provide a flexible and cost-effective alternative [79]. They are applied in a complementary way to satellite platforms and offer a high spatial resolution (SR) of measurement, generally between 2 and 10 cm, for a more accurate stress detection and field validation. Their flexibility gives an advantage in the temporal frequency of data acquisition, which is performed on-demand and which allows for quick phenotyping on different targeted fields [76]. Drones can be also coupled with different sensor types including RGB (visible light), multispectral, hyperspectral, thermal infrared [80], and LiDAR (Light Detection and Ranging) which enable accurate estimation of factors that are significant for monitoring early symptoms of abiotic stress [76].

The satellite-based RS has revolutionized the detection of abiotic stress by providing contactless, large-scale monitoring tools [72,76]. Satellite-based systems are defined by their ability to collect electromagnetic data from the Earth’s surface via sensors placed on orbiting platforms with a SR between 10 and 1000 m [76]. This technology utilizes different satellites platforms including Sentinel-2 with a SR between 20 and 60 m applied in regional water stress and nitrogen status, Landsat 8/9 (NASA) integrating a SR between 15 and 100 m for thermal stress detection, MODIS (NASA) equipped of SR between 250 and 1000 m for continental drought monitoring and ECOSTRESS (NASA) with a RS of 70 m for diurnal plant water stress investigation [72].

### 5.3. Imaging Techniques

Imaging techniques have proven to be reliable in data collection for abiotic stress detection in plants. AI is heavily reliant on data to process and make informed decisions, mimicking human intelligence. In agriculture, visual and numerical data are both key to detecting stress. Imaging sensors help in the collection of visual data such as physical changes in appearance. There are various types of imaging sensors, including RGB, hyper- and multispectral, fluorescence, and thermal imaging sensors [18].

The RGB imaging sensor is most popular and accessible phenotyping technique for abiotic stress detection [18]. It uses visible light to capture plant images in Red, Blue, and Green (RGB). Specifically, blue light is detected between 400 and 499 nm spectral bands, green between 500 and 549 nm of spectral bands and red between 550 and 750 nm of spectral bands in order to identify physiological and structural anomalies on plants [44]. This method facilitates the non-destructive measurement of morphological traits including plant height, biomass, and projected shoot area for monitoring growth rates and yield potential under adverse conditions [13]. In addition to provide structural data, the capacities of RGB sensors extend also to objectively measure and quantify changes in color (colorimetric shifts), such as the leaf yellowing induced by nitrogen deficiency or the accelerated senescence associated with salinity and water stress [13,44]. These sensors are deployed across multiple scales using platforms ranging from handheld smartphones and low-cost onsite devices to high-throughput UAVs for field level monitoring [13]. Despite its high versatility and cost effectivity, RGB sensors are limited to the inability to detect pre-symptomatic biochemical changes and to sensitivity to environmental noise such as ambient illumination and background clutter which can affect image quality and reliability. To address this issue, some review papers have reported the use of ML algorithms and advanced image processing such as normalization (gray-card/white- balance calibration), shadow removal, and leaf background segmentation followed by feature learning, for improving accuracy of RGB sensors in stress detection [44].

Spectral imaging sensors are convenient for stress detection using wavelengths beyond the visible light including multispectral and hyperspectral imaging techniques. They are commonly used to detect subtle changes in plant appearance that may not be captured in visible light [18]. Specifically, hyperspectral imaging has emerged as one of the most powerful sensing technologies for the detection of abiotic stress due to its capacity to capture various wavelengths typically ranging from the visible to the shortwave infrared region (400–2500 nm). Its combination with AI model makes it more interesting during image treatment. In practical, hyperspectral imaging is not used as raw images but undergoes preprocessing techniques before analysis. The preprocessing pipeline starts essentially with the radiometric calibration, often referred to as white and black calibration, which converts raw light intensity into standard reflectance [81,82]; then, the process undergoes image segmentation to isolate the plant tissue as the region of interest by removing irrelevant backgrounds and bad pixels [81]. The data will further undergo spectral signal enhancement which consists of mathematical pre-treatments which help to suppress noise and emphasize subtle stress indicators. Due to the massive amount, high dimensionality, and redundancy of data, hyperspectral data will undergo a dimensionality reduction process to reduce redundancy and improve model efficiency through different techniques such as Principal Component Analysis (PCA) which transforms spectral data into orthogonal variables (principal components) which separate treatment groups based on variances [81]. From the processed data, a diverse range of spectra features are extracted, including photosynthetic pigments, red-edge position shifts, water content, structural and storage compounds and VIs such as NDVI, GNDVI (Green NDVI), MCARI (Modified Chlorophyll Absorption in Reflectance Index), RDVI (Renormalized Difference Vegetation Index), etc. [82]. These features will serve as inputs for machine learning and deep learning models to classify stress type and severity. On the other hand, multispectral imaging captures electromagnetic reflectance in several discrete spectral bands [44] (400–1000 nm), sampling between 4 and 12 bands across the visible, red-edge, and near-infrared (NIR) regions [72]. By integrating these specific wavelengths ranges, this technique facilitates the derivation of diverse VIs such as NDVI, PRI and REIP (Red-Edge Inflation Point), which quantify critical physiological traits to detect nitrogen deficiency, drought impact, and salinity at an early stage [72]. Although it possesses lower spectral resolution than hyperspectral imaging, multispectral sensors are preferred for field-scale monitoring due to it portability and affordability, particularly when deployed on UAVs or handheld platforms [44].

Chlorophyll Fluorescence imaging sensors capture spatially resolved electromagnetic emissions to evaluate the photochemical efficiency and physiological health of plants under abiotic stress [44]. This method functions on the principle that light absorbed by chlorophyll is utilized for photochemistry, dissipated as heat, or re-emitted as chlorophyll fluorescence; thus, measuring the light emitted provides a non-destructive indication of the plant’s current metabolic state [13]. Key parameters, such as the potential quantum yield of photosystem II (Fv/Fm), are particularly effective for identifying pre-symptomatic responses to drought, heat, salinity, and nutrient deficiency often before they manifest in the visible spectrum [13]. Advanced implementations, such as multicolor fluorescence imaging, utilize ultraviolet (UV) excitation to map intensity across discrete spectral bands, typically blue (F440), green (F520), red (F680), and far-red (F740), allowing the detection of photosynthesis disruption across the leaf or canopy. While offering high sensitivity for early stress, this technique requires dark adaptation (often 30 min) to establish stable baseline levels and may be sensitive to specific excitation setups [44].

Thermal imaging sensors are used to detect plant stress by measuring variations in plant temperature. Variations in plant temperature are critical to understanding the effects of water stress or plant diseases. It is most applicable in large-scale applications [4]. This imaging technique utilizes specialized sensors to detect emitted infrared radiation, typically in the range of 8–14 µm, to quantify spatially resolved leaf and canopy temperatures [44,72]. This particular methodology serves as a physiological indicator that can be utilized for the purpose of observing and evaluating stomatal conductance and transpiration rates, as abiotic stresses like drought and extreme heat induce stomatal closure, which subsequently inhibits evaporative cooling and consequently causes a detectable elevation in leaf temperature compared to the ambient air [44]. The outputs are represented as false-color images where specific thermal anomalies and indices, such as crop water index, allow the identification of water deficits before physiological damage becomes visible in the visible spectrum [13]. Like other imaging techniques, the accuracy of this method is highly affected by environmental noise such as wind, humidity and incident radiation, which require serious radiometric calibration, emissivity adjustments, and ambient compensation during preprocessing [44].

**Figure 3:**
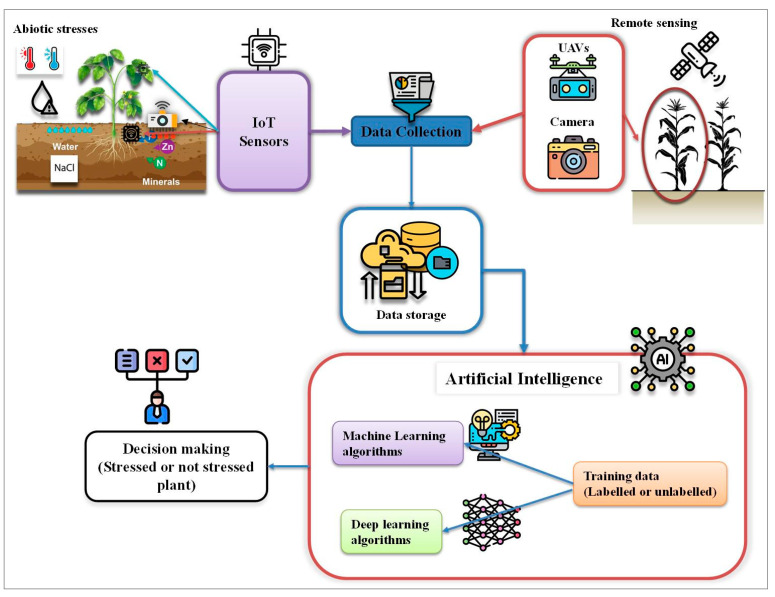
Architectural model of abiotic stress detection tools using artificial intelligence (AI). The figure illustrates a conceptual workflow of an AI-based system for detecting plant stress. The model begins with data collection, in which environmental and plant stress parameters are monitored by various remote sensing devices integrated within an Internet of Things (IoT) network. All collected data are consolidated within a Data Collection module, stored in a Data Storage module, and subsequently subjected to preprocessing step before being transferred to the AI module. The AI module uses machine learning and deep learning algorithms trained to recognize patterns and features associated with both stressed and non-stressed plants. The output of the AI module supports a decision-making process for plant stress detection.

Figure 3 presents the framework steps of abiotic stress detection using AI tools adapted from studies conducted by Jafar et al. [29] and Belmir et al. [53].

## 6. Artificial Intelligence Application for Specific Abiotic Stress Detection

### 6.1. Water Stress Detection

Several recent studies demonstrated significant developments in water stress detection using AI through various technological approaches. In a study conducted by Chandel et al. [83], a comparative study was conducted to evaluate three DL models, AlexNet, GoogLeNet, and Inception V3, to identify water stress in maize, okra and soybean. GoogleNet model showed the highest performance in classifying the images from water and no water stressed with more accuracy (98.30, 97.50 and 94.16% for maize, okra, and soybean crops, respectively) compared to the two other models. Later on, from the same author, Chandel et al. [84], an AI-enabled mobile device was developed to identify water stress in real-time conditions on maize and wheat crops. The device was a portable apparatus which assembles a Raspberry Pi and RGB camera based on GoogLeNet DL model to process captured images and classify them in stress and unstressed categories. In its finds, the model could still perform high classification accuracy of 92.7% and 97.9% in wheat and maize, respectively. In an early conducted study, Soffer et al. [85] proposed a new method for water stress level classification detection based on DL and digital images. In this case, two ways of classification were performed based on a Convolutional Neural Network for spatial feature extraction and a Long Short-Term Memory for temporal features) extraction, which was able to detect, during the day, changes in water stress levels and to classify five different water stress levels with a high accuracy (91.7%). In an advanced research, Rossi et al. [86] developed an accurate and automatic image-based algorithm for identification of plant phenotypic traits such as plant height, petioles inclinations, and leaf area through a 3D-models segmentation for early water stress detection on four tomato varieties. They found that, for some varieties, the height was the earliest and most sensitive sign of water stress.

### 6.2. Drought Stress Detection

ML algorithms coupled with imaging techniques have been used to detect drought stress in plants. ML models, such as random forest and multilayer perceptron neural networks, have been used to analyze thermal images for detecting progressive drought stress in a diverse panel of *Gossypium hirsutum* L. (cotton) genotypes. Data collected from images through trained AI models made it possible to extract features like leaf temperature to classify plants as well-watered or undergoing dry-down. The two trained models performed high classification accuracies with 75% (Random Forest (RF)) and 78% (Multilayer Perceptron (MLP) neural networks) [87]. In a study conducted by Mohammadi and Asefpour Vakilian [88], ML methods, particularly Support Vector Machines optimized by metaheuristic techniques, showed high specificity in detecting salt and drought stress in cucumber plants using morphological, physiological/biochemical, and miRNA characteristics. Under both stress conditions, the coefficient of determination for predicting the stress type and severity in plants was 0.61, 0.82, and 0.99 using morphological, physiological/biochemical, and miRNA characteristics, respectively. Other studies developed a high-throughput by using DL with a trained YOLOv8 model and infrared images to detect drought stress stages in lettuce seedlings. For collecting infrared images, a Raspberry Pi robot was used to facilitate the collection and the result showed a high efficiency in early stress detection in lettuce [89].

### 6.3. Salinity Stress Detection

ML has gained reputable applications in detecting salinity stress in plants. Support Vector Machine (SVM) leverages a genetic algorithm to detect and evaluate the magnitude of salinity stress in plants. Additionally, an electronic nose system is used employing temperature-modulated gas sensors and ML algorithms, specifically SVM and random forest classifiers, in detecting salinity stress in Khasi Mandarin Orange plants by analyzing leaf-emitted volatile organic compounds. The output form this developed technology showed a significant result in salinity stress detection with an achieved accuracy of 98.3% [90]. In other studies, researchers evaluated the power of multicolor fluorescence imaging combined with advanced data analysis, a SVM and principal component for image classification, to detect early salinity stress in the *Arabidopsis* plant. For this concern, a self-developed system was used to collect fluorescence images at various wavelengths. This complex performed a high classification accuracy, leading to the conclusion of strongly recommending these tools for an early salinity stress detection [91].

### 6.4. Heat Stress Detection

The DL algorithm has shown great research improvements in plant heat stress detection. New advanced studies on systems for monitoring apple sunburn susceptibility have been carried out and focused on the introduction a color-independent Mask R-CNN (Region-based Convolutional Neural Network) model for segmenting apple fruit for an early detection. To this end, an IoT- and edge-compute-enabled sensing system was used to estimate fruit surface temperature (FST) with high accuracy. The output validated the Mask R-CNN model, achieving high segmentation accuracy (91.4% average precision) and thus, suggesting a potential tool for actuating a cooling system in orchards [92]. In other recent advancements, researchers have highlighted the use of imaging techniques such as visible, near-infrared, thermal, hyperspectral, and fluorescence imaging used to characterize plant responses under heat stress combining with robotic systems for collecting data and equipped with conventional ML and DL algorithms to identify heat-tolerant traits in plants. This advancement showed more interest in plant breeding area for developing new varieties able to evolve in the changing climate and to ensure future food security [93]. In further studies, more advanced technologies have highlighted heat stress detection combining ML models and IoT technologies. For this purpose, an in situ, ML-enabled subsurface sensor system is trained to learn soil characteristics data to predict the ambient air temperature in order to detect heat stress on crops. The output of this technology may be important in the agriculture sector because it will empower farmers and governments to make early decisions to mitigate negative impacts on crop yield and quality [94].

### 6.5. Nutrient Deficiency Detection

AI has been used to detect nutrient deficiency in plants using artificial neural network classifiers. Jose et al. [95] have employed ML and algorithms to develop an artificial neural network model that can detect nutrient deficiency stress in plants using leaf characteristics. This system uses spectral imaging sensors to capture leaf data, then algorithms assess nutrient deficiency by processing and evaluating the information against existing information. In another study conducted by Jeong et al. [96], a ML algorithm, YOLOv8s, was used to detect nitrogen, phosphorus, and potassium deficiency in soybean plants. The models achieved satisfied performance, with classification accuracy of 99.18% during training and 98.51% for validation. More studies like one conducted by Guntupalli et al. [97] have introduced CoLeafNet, a dual-track DL model for detecting nutrient deficiency in coffee leaves. As stated, it is a novel approach for precision agriculture for nutrient deficiency classification in coffee leaves which showed high accuracy (95.64%) in terms of classification compared to other DL models. Specifically, this model was used to identify Boron, Iron, Potassium, Calcium, Magnesium, and other deficiencies in coffee leaves.

Table 3 summarizes the findings of the AI application in specific abiotic stress detection which provides details regarding specific study focuses by highlighting the targeted plant and study goal, as well as the technologies used for assessment, the AI models used, and the key findings of the AI applications.

### 6.6. Real-World Applications AI in Modern Agriculture

Beyond scientific studies and classification accuracy evaluation, several AI-based detection systems have been implemented in farm operations for practical decision-making. For example, a specific case based on a platform developed by Microsoft, called Microsoft FarmBeats [98], focused on data-driven agriculture for improving farming efficiency, sustainability, and productivity. The model collects a massive amount of information from soil sensors, drones, and weather stations, then uses AI models to generate a more efficient water management schedule and thus avoid abiotic stress like drought damage to occur in the field. The data provided by the sensors, images (multispectral and hyperspectral images for example) from drones combined with a specific trained AI model, can detect anomalies, predict stress, distinguish different stresses (drought, nutrient, and heat stress), and make a stress hotspot map across the agricultural field, which helps to make early decisions for addressing those environmental challenges.

Similarly, other AI-driven platforms modify modern agriculture by integrating diverse real-time data for better management. For example, digital technology companies such as IBM utilize a system called IBM Watson Decision Platform for Agriculture, which applies AI to data coming from weather stations, soil sensors, and satellite imagery to give farmers detailed information on field conditions, resources needed, and help to make decisions [99].

Moreover, a company, Palmaz Vineyards, based in California’s Napa Valley, has adopted AI for daily vineyard management. They use geospatial technology (satellite and drone imagery ) and monitoring systems for guidance and decision-making in their winemaking processes. This company uses a model called VIGOR (Vineyard Infrared Growth Optical Recognition) which helps to manage abiotic stressors like extreme weather, spring frosts, droughts, and heatwaves in the vineyards, which are the primary risks to grape production. For this purpose, researchers have applied Random Forest (RF) and Extreme Gradient Boosting (EGB) algorithms to hyperspectral and sentinel-2 multispectral imagery to quantify the impacts of heatwaves and model water stress on irrigated vineyards [100]. This gives an example of a well-integrated AI model in agricultural farms that helps farmers to manage their vineyards efficiently.

Another American company, named John Deere, specializes in the manufacture of agricultural machinery and has developed autonomous tractors and combine harvesters that integrated sensors that gather information in real time on soil conditions, crop conditions, and the overall field conditions. These machines integrate machine learning algorithms and analyze the collected data, thus enabling farmers to make data-driven decisions regarding irrigation practices, fertilization strategies, and the application of pesticides on crops. The integration of this technology helps to improve yields and has a significant impact on the minimization of negative effects on the environment [26].

## 7. Detection of Abiotic Stress in Outdoor and Indoor Cultivation

AI tools and methods of stress detection can be used both indoors and outdoors. Indoor cultivation environments imply that abiotic factors can be controlled to designated levels convenient for plant survival. In greenhouses, humidity, water availability, temperature, salinity, and nutrient content in plants and within the environment are subject to researchers’ preferences. These environments empower machine and DL models and other AI tools to collect data with high precision and provide accurate results. Imaging techniques such as spectral and fluorescence imaging function better in controlled settings [101]. ML models, such as random forest and gradient boosting, which measure stomatal count and density in crops, provide a high precision of results in indoor settings as compared to outdoor settings. Stable environments within indoor settings foster high precision in AI models and tools [87]. Models applicable in indoor settings offer limited scalability.

Outdoor settings present a real-world scenario. Models and tools applicable in outdoor settings provide high scalability with limited precision due to the complexities of outdoor environments. Factors such as human activities, the presence of diverse plant species, and other data points require integrated approaches. AI tools and methods used in outdoor settings offer multidisciplinary approaches in data collection. This enhances the collection of a variety of sets of data used to confirm different scenarios. Imaging techniques such as satellite imaging encompass all other sensors, and are hence able to collect relevant data from different settings. This allows them to capture physical and biochemical data to recognize and confirm the effects of abiotic factors [18]. Tools used in outdoor environments are built to withstand the effects of increasing pressure and cover larger areas. As such, ground vehicles and UAVs are more suitable for collecting data in large areas within a short time. UAVs used in an outdoor setting combine imaging with IoT data to enhance the precision of results. Models and tools used in outdoor environments are more scalable in real-world settings [102].

## 8. Challenges and the Future of AI in the Detection of Abiotic Stress in Plants

The detection of abiotic stress in plants using AI holds immense potential to transform crop production, yet it also poses significant challenges. High-quality data collection remains one of the most critical steps in AI-based stress detection. Large volumes of data are generated from various RS tools, but they often contain background noise and inconsistencies that threaten their integrity. Further developments in technology and AI result in the availability of even more complex data. There are no clear data annotation instructions. Inconsistency in data annotation limits the exploration of significant insights hidden in complex data. Compromised data quality impedes further research on the application of AI in the detection of abiotic stress in plants [103].

There are concerns about the sustainability of AI-based stress detection models in agricultural fields due to harsh environmental conditions. Extreme weather conditions, such as heavy rainfall and extreme temperatures, may cause devices to degrade over time. Replacement of degraded devices increases the risk of e-waste pollution and increased operational costs, which may not be attainable for low-income farmers. Limited exposure coupled with the high cost of investment in AI infrastructure is a major barrier to adoption among low-income farmers. Most farmers in developing countries are stuck in traditional methods of farming; hence, limited exposure to AI, coupled with the financial burden, makes AI expensive to adopt [104]. Another drawback lies in data privacy and security. The introduction of AI stress detectors has resulted in farmers generating massive data on their farms without their knowledge. There are no clear regulations on ownership and privacy of data [104]. This situation exposes farmers to risks of cyber-attacks, illegal surveillance, and data breaches, which bear economic and data losses [105].

The future of AI in stress detection in plants bears significant benefits in agriculture. It involves the improvement in degrees of precision in AI models and tools. This involves imaging techniques with high-resolution features. The next phase of agriculture requires improved precision and timing in detecting stress in plants to enhance timely interventions. It also involves the development of devices that work optimally with minimal energy requirements. It is imperative to enhance adoption in low and middle-income countries with relatively minimal progress in energy sectors as compared to developed countries [104]. Next-generation AI stress detection equipment should be empowered to automatically adapt to changing environmental patterns through self-learning mechanisms. Emerging trends, such as blockchain technology, have the potential to tackle trending challenges of AI stress detection in plants. There is an opportunity to improve plant stress detection by integrating AI solutions with genome editing to improve the quality of plants [106]. The future of AI in agriculture is anchored on global multidisciplinary approaches [104].

## 9. Conclusions

AI is gradually revolutionizing agriculture across multiple domains, including abiotic stress detection. It leverages data collected through various sources, including imaging techniques and sensors, to develop and train systems that emulate human thinking and intelligence. These systems rely on tools such as ML and DL to process large datasets into actionable insights. AI-based approaches provide more accurate and effective methods of detecting stress in plants through multimodal data-driven decision-making. Key aspects of AI, such as data analytics, predictive modeling, and real-time monitoring of abiotic stressors in plants, help farmers to make informed management decisions that reduce losses, optimize resource use, and increase yields. These methods are also valuable to the development of stress-resilient cultivars adaptable to changing environmental conditions. However, revolutionary AI technology is not without drawbacks. In developing countries, key barriers to the adoption of AI include limited access to AI tools, inadequate technical expertise, and financial constraints. Lack of a well-defined governance framework for managing data collected through AI models and tools exposes farmers to risks of data breaches and cyber-attacks, potentially resulting in economic and data losses. Addressing these challenges requires the establishment of robust data protection policies, improvement in algorithmic precision, reduction in energy demands, and adoption of emerging technologies such as blockchain technology to foster data protection, transparency, and trust.

## Figures and Tables

**Table 1:** Comparative table between traditional methods and AI-based technologies in abiotic stress detection.

Features	Traditional Methods	AI-Based Technologies
Detection timing	Detect stress only after symptoms start to appear, delaying important or necessary interventions	Allow early detection of pre-symptomatic stress by capturing subtle phenotypic changes, which is relevant for AI models to identify stress before it fully manifests
Precision and Accuracy	Relatively subjective and relies on the personal judgment and perception of the person doing the scouting; limited to observable elements using human eye in visible light. Lab tools offering high accuracy	Accuracy based on the use of data-driven decisions; progressively improved with more high-quality data
Scalability	Difficult to scale detection application on a large area	Provide high scalable solutions (e.g., Using UAVs for satellite imagery)
Labor efficiency	Intensive labor and time-consuming activity; intensity of labor increases with land size	Improve operational efficiency through automation and non-destructive monitoring
Disadvantages/Costs	Destructive sampling required for laboratory analysis which incurs analytical costs	AI models require large high quality labeled datasets; acquisition and maintenance of AI tools such sensors and supported platforms increase the operational costs

**Table 2:** IoT-enabled sensors used for abiotic stress detection in plants.

Sensor Type	Parameter(s)	Target Stress	Reported Accuracy	Advantages	Limitations	References
Soil moisture sensor	Volumetric water content	Water	*R*^2^ = 0.87 [51]	Real-time monitoring, low cost, efficient, accurate and reliable,	Requires calibration, variability in accuracy depending on the type of soil, limited scalability, sensor installation, external power requirement	[51,52,53]
Soil temperature sensor	Soil temperature	Heat	NA	Real-time monitoring, low cost, better repeatability in temperature measurements, higher accuracy, better stability	Nonlinearity and calibration complexity, environmental sensitivity	[54]
Soil EC sensor	Electric Conductivity	Nutrient deficiency, salinity	*R*^2^ > 0.85 for K^+^ and *R*^2^ > 0.86 for Ca^2+^ [55]	Real-time monitoring, high sensitivity to nutrient variations, fast data acquisition, low cost	Requires calibration, high sensitivity to soil moisture, lack of specificity, crop residue interference	[55,56,57]
Ion-selective electrode	Essential nutrients	Nutrient deficiency	NA	High accuracy, low detection limits, real-time monitoring, low cost	Environment interference, lack of integration, dependence on laboratory equipment	[58,59]
Sap flow sensor	Sap flow rate, transpiration rate	Drought, water	Accuracy = 77% [60]	Real-time monitoring, early stress detection, non-invasive monitoring	Invasiveness and wounding response, complexity of calculation, high cost	[60,61,62,63]
Infrared temperature sensor	Temperature and emitted radiation	Drought, water, heat	NA	Low cost, non-destructive, real-time monitoring, high temporal resolution	Limited scalability (point measurement), requires careful calibration, high environmental sensitivity	[22,64,65]
Chlorophyll fluorescence sensor	Chlorophyll fluorescence (Fv/Fm)	Drought, heat, cold, salinity, nutrient deficiency	NA	Simple, early stress detection, non-destructive, low cost, rapid, high sensitivity	Complex interpretation, restrictive requirements	[23,66,67,68]
RGB imaging	Canopy morphology, Basic color channels (RGB)	Drought, salinity, nutrient deficiency	*R*^2^ ≥ 0.80 [69]	Low cost, availability, versatility and trait measurement	Spectral and detection limitation, need for stable calibration, image interpretation challenge	[13,69,70]
Thermal imaging	Canopy temperature (°C)	Drought, water, heat, nutrient deficiency	Accuracy > 75% [22]	Spatial data, high scalability, non-destructive non-destructive, early stress detection, flexibility and accessibility	Sensitive to environmental factors, need for corrections and calibration, complex computational requirements	[22,71]
Multispectral imaging	Reflectance (400–1000 nm), VIs (NDVI, NDRE, etc.)	Drought, water, Salinity, heat, nutrient deficiency	NA	Fast data acquisition, high spatial coverage and scalability, early stress detection, affordability relative to hyperspectral imaging, operational simplicity	Limited spectral resolution, requires advanced processing	[13,20,72]
Hyperspectral imaging	Spectral parameters (400–2500 nm), VIs (NDVI, NDRE, etc.), chlorophyll degradation, water content	Drought, water, Salinity, heat, nutrient deficiency	*R*^2^ > 0.86 [73]	Ultra-high spectral resolution, early stress detection, advanced stress discrimination	Complex data processing, high cost, environmental interference, redundancy and noise	[73,74,75]

EC = electrical conductivity; NDVI = normalized difference vegetation index; NDRE = normalized difference red edge; NA = not available; VIs = Vegetation Indices.

**Table 3:** Summary table of AI applications for specific abiotic stress detection.

Stress Type	Study Focus (Plant/Goal)	Technology and Data Source	AI Models	Key Results and Accuracy
Water stress	Maize, Okra and Soybean	Comparative study based on digital images	GoogLeNet (DL model)	Highest performance: Maize (98.30%), Okra (97.50%), Soybean (94.16%) accuracy
	Maize, Wheat	AI-enabled mobile device (Raspberry Pi, RGB camera)	GoogLeNet (DL model)	High classification accuracy: Maize (97.9%) and Wheat (92.7%)
	Corn	Digital images	CNN (Spatial) + LSTM (Temporal) (DL models)	High accuracy (91.7%) in classifying five different water stress levels
	Tomato varieties	Automatic image analysis	Image-based algorithm (3D-models segmentation)	Height was found to be the earliest and most sensitive sign of water stress
Drought stress	Cotton genotypes	Thermal imaging	Random Forest, multilayer Perceptron neural networks (ML models)	High performance Random Forest (75%) and multilayer Perceptron neural networks (78%) accuracy in the treatment prediction
	Cucumber	Image textural features (Morphology), physiological/biochemical miRNA characteristics	Support Vector Machine (ML model)	The Morphological data yielded a 0.61 coefficient, physiological/biochemical gave 0.82, and miRNA characteristics resulted in 0.99 coefficient for stress prediction
	Lettuce seedlings	High-throughput system using infrared images (Raspberry Pi robot)	Trained YOLOv8 model (DL model)	High efficiency (93.62%) in early stress detection
Salinity stress	Khasi Mandarin Orange plants	Electronic nose system (analyzing leaf-emitted volatile organic compounds)	SVM and Random Forest classifiers	High performance in stress detection with 98.3% of accuracy
	Arabidopsis plant	Multicolor fluorescence imaging (self-developed system)	SVM and Principal Component	Performed high accuracy classification for early detection in salt-stressed plants
Heat stress	Apple fruit	IoT- and edge-compute-enabled sensing system (Estimating Fruit Surface Temperature—FST)	Mask R-CNN	Achieved high segmentation accuracy (91.4% average precision), suggesting use for actuating cooling systems
	Characterize plant responses/identify heat-tolerant traits	Various imaging techniques (visible, thermal, hyperspectral) combined with robotic systems	Conventional ML and DL algorithms	Advancement for use in plant breeding area
	Prediction of air ambient temperature	IoT technologies (learning soil characteristics)	ML-enabled subsurface sensor system	Results are important for farmers/governments to make early mitigation decisions
Nutrient deficiency	Detecting nutrient deficiency stress using leaf characteristics	Spectral imaging sensors	Artificial Neural Network	Assesses deficiency by processing and evaluating leaf data
	Soybean plants (detecting N, P, K deficiency)	Classification model	YOLOv8s (ML algorithm)	Achieved satisfied performance with a validation accuracy of 98.51%
	Coffee leaves (detecting B, Fe, K, Ca, Mg, etc.)	Classification model	CoLeafNet (dual-track DL model)	Showed high accuracy of 95.64% of classification

## Data Availability

Not applicable.

## Abbreviations

The following abbreviations are used in this manuscript:

AI	Artificial Intelligence
ANN	Artificial Neural Networks
CNNs	Convolutional Neural Networks
CWSI	Crop Water Stress Index
DL	Deep Learning
DNNs	Deep Neural Networks
DQN	Deep Q-Networks
EC	Electrical Conductivity
EVI	Enhanced Vegetation Index
Fv/Fm	Chlorophyll fluorescence (variable fluorescence/maximum fluorescence)
GANs	Generative Adversarial Networks
GNDVI	Green Normalized Difference Vegetation Index
IoT	Internet of Things
k-NN	k-Nearest Neighbors
LST	Land Surface Temperature
MCARI	Modified Chlorophyll Absorption in Reflectance Index
ML	Machine Learning
NA	Not Available
NDRE	Normalized Difference Red Edge
NDVI	Normalized Difference Vegetation Index
NDWI	Normalized Difference Water Index
PCA	Principal Component Analysis
PRI	Photochemical Reflectance Index
REIP	Red-Edge Inflation Point
RDVI	Renormalized Difference Vegetation Index
RF	Random Forest
RGB	Red, Green, and Blue (spectral bands/imaging)
RNNs	Recurrent Neural Networks
RS	Remote Sensing
SPAD	(Type of meter used to measure relative chlorophyll content)
SVM	Support Vector Machines
TD	Temporal Difference
UAVs	Unmanned Aerial Vehicles
VIs	Vegetation Indices
